# A global survey on the utilization of cryotherapy and compression therapy for the prevention of chemotherapy-induced peripheral neuropathy

**DOI:** 10.1007/s00520-022-07383-x

**Published:** 2022-10-10

**Authors:** Alexandre Chan, Amna Elsayed, Ding Quan Ng, Kathryn Ruddy, Charles Loprinzi, Maryam Lustberg

**Affiliations:** 1 Department of Clinical Pharmacy Practice, School of Pharmacy and Pharmaceutical Sciences, University of California Irvine, Irvine, CA USA; 2 Department of Pharmacy, University of California Irvine Health, Irvine, CA USA; 3 Department of Oncology, Mayo Clinic Alix School of Medicine, Rochester, MN USA; 4 Breast Medical Oncology, Yale Cancer Center, New Haven, CT USA

**Keywords:** Chemotherapy-induced peripheral neuropathy, MASCC, Cryotherapy, Compression therapy, Cancer, Supportive care

## Abstract

**Background:**

Chemotherapy-induced peripheral neuropathy (CIPN) is a serious side effect that is highly prevalent among cancer patients undergoing chemotherapy. There is a growing use of cryotherapy (CryTx) and compression therapy (ComTx) to prevent CIPN at cancer centers worldwide. In this study, we examined the awareness and recommendation of these modalities and evaluated factors associated with awareness. In addition, we investigated the type of technology utilized, barriers to implementation, and perceived adverse events of CryTx and ComTx.

**Methods:**

Active members of the Multinational Association of Supportive Care of Cancer (MASCC) were invited to complete an electronic survey that was sent via SurveyMonkey between September and October 2021. The survey assessed participants’ awareness, recommendation, usage, barriers to utilization, and perceived adverse events of CryTx and ComTx. Descriptive statistics and multiple logistic regression were utilized to analyze findings.

**Results:**

Out of 184 participants, 70.1% were physicians, 73.4% had over 10 years of practice, and 49.5% were practicing in an outpatient setting. While more than half (63.3%) of participants indicated awareness of CryTx for taxane-induced peripheral neuropathy, less than a quarter (22.8%) indicated recommendation in their practice setting. Factors associated with higher awareness of CryTx for patients receiving taxanes include living in Europe (OR = 2.69, 95% CI [1.28–5.64], *p* = 0.009), not practicing in an inpatient setting (OR = 3.15, 95% CI [1.45–6.85], *p* = 0.004), and self-identifying as non-physician (OR = 2.40, 95% CI [1.03–4.37], *p* = 0.041). Commercial cooling (31.5%) and compression (16.8%) gloves and socks were the most used modalities for CryTx and ComTx, respectively. The most identified barriers to CryTx and ComTx utilization include insufficient evidence (53.5%), logistics (34.8%), and patient discomfort (23.4%). Redness/irritation of skin (27.7%) and numbness/tingling (24.5%) accounted for about half of the perceived adverse events associated with use of CryTx and ComTx.

**Conclusion:**

Results of our global survey illustrated that there are varying modes in the delivery of CryTx and ComTx among cancer centers around the world. Education of the utilization of CryTx and ComTx, in addition to efficacy and implementation studies, is needed to close the gap between awareness and implementation in clinical practice.

**Supplementary Information:**

The online version contains supplementary material available at 10.1007/s00520-022-07383-x.

## Introduction

Chemotherapy-induced peripheral neuropathy (CIPN) is a serious side effect that is highly prevalent among cancer patients undergoing chemotherapy. It can lead to unwanted symptoms such as numbness and paresthesia which can reduce quality of life and cause treatment delay and/or discontinuation [1]. Several chemotherapy agents are known to be associated with CIPN; these agents include taxanes (e.g., paclitaxel), platinum agents (e.g., oxaliplatin), and vinca alkaloids (e.g., vincristine) [2]. Currently, guidelines are available for managing CIPN caused by paclitaxel and oxaliplatin [3, 4]. However, there is a lack of effective strategies for the prevention of CIPN caused by these agents. The American Society of Clinical Oncology (ASCO) guidelines recommend assessing the risk and benefit of agents known to cause CIPN in patients with underlying neuropathy and those with comorbidities that predispose them to neuropathy [3]. Moreover, the guidelines indicate that cryotherapy and compression therapy may prevent CIPN symptoms, but due to limited studies, make no recommendation regarding their utilization outside of clinical trials [3]. Other guidelines, such as the European Society of Medical Oncology (ESMO) guidelines, state that cryotherapy and compression therapy can be considered preventive strategies for CIPN [4]. Although there are studies in the literature that assess the efficacy of these modalities, they are limited and most have small sample sizes [5–7]. Moreover, few studies were designed to compare the efficacy of these modalities to one another [8].

Cryotherapy refers to the cooling of the extremities by administering cold therapy (icepacks, wearable garments, etc.) while the patient is receiving chemotherapy [1, 3, 4]. Compression therapy, on the other hand, refers to wearing stockings or gloves to reduce the microvascular flow while the patient is receiving chemotherapy [1, 3, 4]. It is thought that the vasoconstrictive effect of these methods will limit the local effect of chemotherapy, thus preventing chemotherapy toxicity in the extremity nerves. These novel preventive strategies are being used at some centers in an attempt to prevent CIPN caused by taxane and oxaliplatin, which are most well known for their ability to cause CIPN [3].

Over the past decade, several studies were published to assess the efficacy and safety of various cryotherapy and compression therapy in preventing CIPN [5–7]. However, there is a lack of literature investigating the awareness and degree of implementation of these methods in clinical settings. Thus, the current study was designed to characterize the current practices of how cryotherapy and compression therapies are used. The objective of this research was to examine the awareness and the prevalence of recommendations regarding cryotherapy and compression therapy among oncology healthcare professionals. In addition, we sought to investigate the type of cryotherapy and compression therapy technology utilized, factors associated with its use, barriers for implementing these modalities in clinical practice, and perceived adverse events.

## Methods

### Study design

This is a cross-sectional survey administered to active members of the Multinational Association of Supportive Care of Cancer (MASCC), a global society of healthcare professionals and researchers in cancer supportive care. This study was exempted by University of California Irvine Investigational Review Board, and a waiver of informed consent was obtained.

### Participant eligibility criteria

All active MASCC members were eligible and received an invitation to the survey via email. We excluded survey responses with incomplete responses and those completed by participants who did not self-identify as clinicians caring for patients receiving neurotoxic chemotherapy.

### Data collection

Investigators of this study (A.C., M.L., K.R., C.L.) drafted the survey questions which were then assessed for appropriateness by clinicians in the field, prior to circulation (Appendix). The 19-question survey was built electronically using SurveyMonkey. The survey was divided into two parts, with the first part consisting of four demographic questions and the second consisting of 15 questions in four different sections: (i) the first section (five items) inquired about awareness of data suggesting that cryotherapy and compression can be used in patients receiving taxane-based and oxaliplatin-based chemotherapy; (ii) the second section (four items) asked if participants or others in their practice recommend cryotherapy and compression therapy in patients receiving taxane and oxaliplatin; (iii) the third section (four items) asked about the type of technology used to deliver these modalities and the duration of their administration; (iv) the last section (two items) assessed barriers to usage and perceived adverse effects associated with these modalities. The survey contained multiple choice questions supplemented with open-ended responses for participants to provide additional responses. The electronic survey was sent to active members of MASCC via email on September 27, 2021, and was open for 1 month. Two follow-up reminders were sent at the 2-week and 4-week marks.

### Study endpoints

The primary endpoint was the proportion of participants who were aware of the effectiveness of cryotherapy and compression therapy for the prevention of taxane- and oxaliplatin-induced peripheral neuropathy. Secondary endpoints included characteristics associated with cryotherapy and compression therapy awareness, the proportion of participants and their colleagues who recommended cryotherapy and compression therapy at their practice setting, the type of cryotherapy and compression therapy technology used, characteristics associated with the type of technology utilized, the duration of usage before and after chemotherapy administration, barriers to utilization and implementation, and perceived adverse events of cryotherapy and compression therapy.

### Statistical analysis

Descriptive statistics was used to summarize responses to each item. Categorical data is presented as counts and percentages. The chi-square test or Fisher’s exact test was conducted for cross-sectional analyses to determine univariate associations between participant characteristics and awareness as well as types of technology utilized and barriers to implementation. A multivariable logistic regression model was subsequently performed to evaluate participant characteristics associated with higher awareness of cryotherapy and compression therapy. A two-sided *p* value < 0.05 was considered significant. All statistical analysis was conducted using SPSS version 28.

## Results

### Study and participant characteristics

The survey was sent to 2480 MASCC members and a total of 218 (8.8%) members responded. Twenty-eight responses were excluded due to incomplete survey sections and an additional six responses were excluded due to participants not self-identifying as clinicians caring for patients receiving neurotoxic chemotherapy, leaving 184 responses eligible for analysis. Overall, 85% of the members who responded were from Asia (33.2%), Europe (31.0%), and the USA (21.2%). Almost three-quarters of the participants were physicians (70.1%), 73.4% had more than 10 years of practice, and about half (49.5%) were practicing in an outpatient setting (Table 1). When questioned about the type of patients seen in their practice setting, the majority (73.4%) reported that they saw patients receiving both taxane and oxaliplatin chemotherapy in their practice settings.

**Table 1:** Participant demographics (*N* = 184)

Demographics	% (*n*)
Professional role	
Physician	70.1% (129)
Advanced practice provider (NP, PA)	10.9% (20)
Nurse	10.9% (20)
Pharmacist	5.4% (10)
Physical therapist	2.2% (4)
Dietitian	0.5% (1)
Years of practice	
More than 10 years	73.4% (135)
6–10 years	20.1% (37)
Less than a year	5.4% (10)
1–5 years	1.1% (2)
Location of practice	
Asia	33.2% (61)
Europe	31% (57)
USA	21.2% (39)
Australia	5.4% (10)
Canada	3.3% (6)
South America	2.2% (4)
Africa	1.6% (3)
Central America	1.1% (2)
Middle East	1.1% (2)
Type of practice setting	
Outpatient oncology	49.5% (91)
Inpatient/hospital	21.2% (39)
University/research	25.5% (47)
Both inpatient and outpatient	3.3% (6)
Home health	0.5% (1)

### Awareness of effectiveness of cryotherapy and compression therapy

Approximately two-thirds (63.3%) of the participants indicated awareness of data suggesting that cryotherapy might decrease taxane-induced CIPN while 39.7% indicated awareness of data suggesting that compression therapy might decrease the effect of taxane-induced CIPN. Thirty-one percent indicated awareness of a combination of cryotherapy and compression therapy to decrease the effect of taxane-induced CIPN. In addition, 31% of participants indicated awareness of cryotherapy being used with patients receiving oxaliplatin (Table 2).

**Table 2:** Participant awareness and recommendation of cryotherapy and compression therapy (*N* = 184)

	Taxane			Oxaliplatin		
	Cryotherapy	Compression	Both	Cryotherapy	Compression	Both
Awareness	63.6% (117)	39.7% (73)	31% (57)	31% (57)	N/A	N/A
Participant recommends	22.8% (42)	1.6% (3)	11.4% (21)	7.1% (13)	4.3% (8)	4.9% (9)
Colleague recommends	16.8% (31)	2.2% (4)	8.2% (15)	4.9% (9)	1.6% (3)	6% (11)

Factors associated with higher awareness of cryotherapy for patients receiving taxane chemotherapy include living in Europe (OR = 2.69, 95% CI [1.28–5.64], *p* = 0.009), not practicing in an inpatient setting (OR = 3.15, 95% CI [1.45–6.85], *p* = 0.004), and self-identifying as non-physician (OR = 2.40, 95% CI [1.03–4.37], *p* = 0.041) (Table 3). There were no significant predictors of awareness of cryotherapy for patients receiving oxaliplatin chemotherapy (Table S1). Similarly, there were no significant predictors of awareness of compression therapy for patients receiving taxane chemotherapy (Table S1).

**Table 3:** Logistic regression model for characteristics associated with awareness of cryotherapy for prevention of taxane-induced peripheral neuropathy (*N* = 184)

	OR	95% CI	*P*-value
Location: Europe	2.686	1.279–5.641	0.009
Setting: non-inpatient	3.154	0.146–0.690	0.004
Profession: non-physician	2.398	0.229–0.970	0.041

### Recommending cryotherapy and compression therapy to patients

Approximately one-quarter (22.8%) of participants indicated recommendation of cryotherapy for taxane chemotherapy while only 7.1% indicated recommendation for oxaliplatin chemotherapy. 16.8% of participants indicated that their colleagues recommended cryotherapy for patients receiving taxane chemotherapy while 4.9% indicated that their colleagues recommended cryotherapy for patients receiving oxaliplatin chemotherapy (Table 2).

1.6% of participants indicated recommendation of compression therapy for taxane chemotherapy and 4.3% for oxaliplatin chemotherapy. 2.2% of participants indicated that their colleagues recommend compression therapy for patients receiving taxane chemotherapy and 1.6% indicated that their colleagues recommend cryotherapy for patients receiving oxaliplatin chemotherapy (Table 2).

11.4% and 4.9% indicated that they recommend both cryotherapy and compression therapy for patients receiving taxane and oxaliplatin chemotherapy, respectively. 8.2% indicated that their colleagues recommend both modalities for patients receiving taxane while 6% indicated that their colleagues recommend both modalities for patients receiving oxaliplatin (Table 2).

### Technology and administration of cryotherapy and compression therapy

Commercial cooling gloves and socks (31.5%) and compression gloves and socks (16.8%) were the most commonly used modalities for cryotherapy and compression therapy, respectively (Table 4). 3.3% of participants indicated using surgical gloves that were a size smaller as a form of compression therapy. In Europe, 40.5% of participants used commercial gloves and socks while 8.3% used bags with ice (*p* = 0.005). In comparison, 29.2% of participants used bags of ice while 9.5% used commercial gloves and socks (*p* = 0.082) in the USA (Table S2).

**Table 4:** Mode and duration of cryotherapy and compression therapy (*N* = 184)

	% (*n*)
Cryotherapy	
Bag with ice	21.7% (40)
Commercial cooling gloves and socks	31.5% (58)
Hilotherm	1.1% (2)
Compression therapy	
Compression gloves and socks	16.8% (31)
Compression bandaging	9.2% (17)
Surgical gloves	3.3% (6)
Manual therapy	0.5% (1)
Device	0.5% (1)
Time before administration	
15 min	2.7% (5)
Half an hour	17.4% (32)
1 h	4.3% (8)
Time after administration	
15 min	2.7% (5)
Half an hour	13% (24)
1 h	6% (11)

17.4% of participants initiate cryotherapy and compression therapy half an hour before chemotherapy administration, 4.3% 1 h prior, and 2.7% 15 min prior (Table 4). Thirteen percent continued cryotherapy and compression therapy half an hour after chemotherapy administration, 6% for 1 h, and 2.7% for 15 min.

### Barriers to cryotherapy and compression therapy utilization

**Fig. 1:**
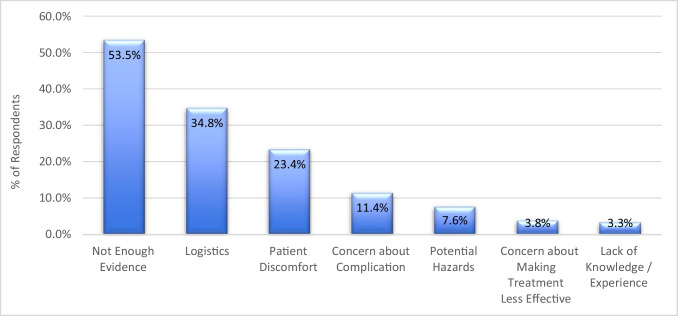
Barriers to utilization of cryotherapy and compression therapy (*N* = 184)

The most identified barriers to cryotherapy and compression therapy usage include insufficient evidence for efficacy (53.5%), logistics (34.8%), and patient discomfort (23.4%) (Fig. 1). Other barriers include concern for complications (11.4%), potential hazards such as ice melting causing slips and falls (7.6%), concern about making treatment less effective (3.8%), and lack of knowledge (3.3%). There were no significant predictors between participant demographics and barriers to utilization for insufficient evidence and logistics (Table S3).

### Perceived adverse events associated with cryotherapy and compression therapy

**Fig. 2:**
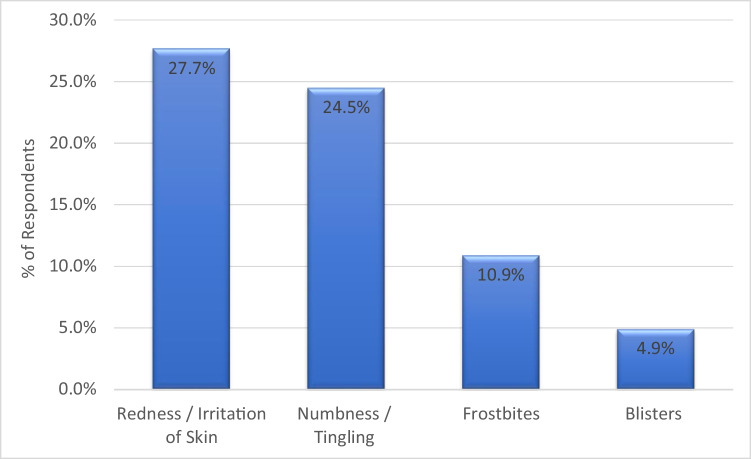
Perceived adverse events associated with use of cryotherapy and compression therapy (*N* = 184)

Redness/irritation of skin (27.7%) and numbness/tingling (24.5%) accounted for about half of the perceived adverse events associated with the use of cryotherapy and compression therapy (Fig. 2). 10.9% and 4.9% noted frostbites and blisters as perceived adverse events, respectively.

## Discussion

This study investigated the awareness and utilization of cryotherapy and compression therapy for taxane- and oxaliplatin-induced peripheral neuropathy. Overall, there was relatively high awareness and recommendation of cryotherapy compared to compression therapy among participants. Awareness for the use of cryotherapy for taxane-induced peripheral neuropathy was highest among participants living in Europe, identifying as non-physicians, and not practicing in an inpatient setting. The higher awareness of cryotherapy can be attributed to the multiple published studies investigating cryotherapy compared to compression therapy [5, 9, 10]. Very few studies compared the efficacy of cryotherapy versus compression therapy for the prevention of CIPN. One study compared the efficacy of cryotherapy using frozen gloves and compression therapy using surgical gloves in preventing paclitaxel-induced peripheral neuropathy [8]. The investigators found no significant difference in incidences of paclitaxel-induced peripheral neuropathy using cryotherapy or compression therapy.

Our results demonstrated that there are varying modes and methods in the delivery of cryotherapy and compression therapy among cancer centers around the world. Commercial cooling gloves and socks were the most used cryotherapy modality in Europe and bags with ice was the most used modality in the USA. Although compression therapy was not as commonly recommended as cryotherapy, compression gloves and socks were the most used compression therapy modality. Notable compression therapy methods include tight surgical gloves which provide a potentially more feasible and less costly option. There are limited studies that compared the efficacy of one modality versus the other for the prevention of CIPN. One study compared the efficacy of surgical gloves for compression therapy for the prevention of paclitaxel-induced peripheral neuropathy compared to bare hands [11]. The study found that the occurrence rate of sensory and motor peripheral neuropathy was lower in the surgical glove hand compared to the control hand (sensory neuropathy 21.4 vs. 76.1%; motor neuropathy 26.2 vs. 57.1%) [11].

The survey data indicated that most participants initiate cryotherapy and compression therapy half an hour before chemotherapy administration and most continue for half an hour after the end of infusion. Most studies in the literature have initiated therapy 15 min before and continued 15 min after the end of infusion for cryotherapy [5–8, 10, 12, 13]. Two studies investigating compression therapy both initiated half an hour before and continued half an hour after the end of infusion [8, 11]. The shorter administration time of cryotherapy compared to compression therapy can be attributed to the increased risk of adverse events including discomfort with cooling. Additional factors may include infusion center chair time for these modalities in addition to the chemotherapy infusion time. This increased chair time may result in a reduction of the number of patients that a center is able to accommodate. Hence, future studies should investigate the need for administration of these modalities prior to and post-chemotherapy administration as well as the efficacy of shorter durations of administration.

The most identified adverse events associated with the use of cryotherapy among our participants included redness and irritation of skin. Overall, studies in the literature noted minimal adverse events associated with the use of these modalities including redness and irritation of skin [5, 13, 14]. Perceived adverse events identified in our study are higher compared to those found in the literature. Ruddy et al. reported 14% of patients experienced some discomfort including numbness/tingling and redness/irritation of skin while our study reported 52% [5]. Although one-tenth of our participants indicated frostbite as an adverse event, current published studies in the literature have not reported such an adverse event.

While almost three-quarter of participants indicated awareness of cryotherapy for taxane-induced peripheral neuropathy, only one-quarter indicated recommendation in their practice setting. Insufficient evidence was the most identified barrier to implementation of cryotherapy and compression therapy. Current studies in the literature show mixed results regarding efficacy of cryotherapy. Additional phase III, randomized, controlled trials are needed to better validate safety and efficacy. Implementation studies are also needed to investigate the application of these modalities in clinical practice. The concept for such a trial has been approved by the United States National Cancer Institute; hopefully, this will clarify the benefits and risks of compression therapy versus cryo-compression therapy versus a control group.

This study had numerous strengths. This was a global survey of supportive care providers and included healthcare providers from various professions and practice settings. Although this study was not designed to assess the efficacy and safety of cryotherapy and compression therapy, it provides a novel perspective to the literature regarding the providers’ opinion of these modalities in clinical practice. This study was limited to MASCC members and response rate was low at 8.8% and, thus, may not represent views of all members. Higher awareness of cryotherapy and compression may also be a reflection of the increased knowledge and interest of MASCC members about cancer supportive care practices relative to non-members.

## Conclusion

This study illustrated that the majority of survey participants are aware of the option of cryotherapy for the prevention of CIPN. Due to the limitations of the currently available clinical trial data, the recommendation for utilization cryotherapy in clinical practice is limited. A large prospective randomized study is currently under development, which hopefully will define the true value of cryotherapy and/or compression therapy for diminishing taxane-induced peripheral neuropathy. Given the high burden of CIPN in the growing number of cancer survivors, additional prevention and therapeutic strategies are needed.

## Supplementary Information

Below is the link to the electronic supplementary material.

Supplementary file1 (PDF 354 KB)

## Data Availability

Data is available as requested.
