# Supplementary material for: A global survey on the utilization of cryotherapy and compression therapy for the prevention of chemotherapy-induced peripheral neuropathy
Source: Support Care Cancer. 2022 Oct 10;30(12):10001–7. doi: 10.1007/s00520-022-07383-x (PMC9715474; doi:10.1007/s00520-022-07383-x)
Supplement: Supplementary file 1 — Supplementary file1 (PDF 354 KB) [file 520_2022_7383_MOESM1_ESM.pdf]

Supplemental Tables

Table S1: Cryotherapy and Compression Therapy Awareness (N=184)

Demographics		Awareness of Cryotherapy for Taxanes (117)	P Value	Awareness of Compression Therapy for Taxanes (73)	P Value	Awareness of Cryotherapy for Oxaliplatin (57)	P Value
**Professional Role**							
Physician	Yes	58.9% (76)	0.046	43.4% (56)	0.077	30.2% (39)	0.731
	No	74.5% (41)		30.9% (17)		32.7% (18)	
Nurse	Yes	75% (15)	0.330	30% (6)	0.469	25% (5)	0.618
	No	62.2% (102)		40.9% (67)		31.7% (52)	
**Years of practice**							
More than 10 years	Yes	63% (85)	0.863	39.3% (53)	0.866	70.4% (95)	0.589
	No	65.3% (32)		40.8% (20)		65.3% (32)	
**Location of practice**							
Europe	Yes	75.4% (43)	0.031	52.6% (30)	0.22	64.9% (37)	0.491
	No	58.3% (74)		33.9% (43)		70.9% (90)	
USA	Yes	64.1% (25)	1.000	30.8% (12)	0.269	64.1% (25)	0.444
	No	63.4% (92)		42.1% (61)		70.3% (102)	
**Type of Practice Setting**							
Inpatient/Hospital	Yes	46.2% (18)	0.010	30.8% (12)	0.269	76.9% (30)	0.250
	No	68.3% (99)		42.1% (61)		66.9% (97)	

Table S2: Usage of Bag with Ice vs Commercial Gloves/Socks across Demographics (N=66)

Demographics		Bag with Ice (n=24)	Commercial Gloves/Socks (n=42)	P Value
**Professional Role**				
Physician	Yes	58.3% (14)	52.4% (22)	0.798
	No	41.7% (10)	47.6% (20)	
**Years of practice**				
More than 10 years	Yes	70.6% (17)	69% (29)	1.000
	No	29.2% (7)	31% (13)	
**Location of practice**				
Europe	Yes	8.3% (2)	40.5% (17)	0.005
	No	91.7% (22)	59.5% (25)	
USA	Yes	29.2% (7)	9.5% (4)	0.082
	No	70.8% (17)	90.5% (38)	

Table S3: Barriers to Usage: Logistical Issues & Not Enough Evidence (N=184)

Demographics		Logistics Issues (n=64)	P Value	Not Enough Evidence (n=98)	P Value
**Professional Role**					
Physician	Yes	38% (49)	0.109	55% (71)	0.520
	No	27.3% (15)		49.1% (27)	
**Years of practice**					
More than 10 years	Yes	31.9% (43)	0.220	56.3% (76)	0.185
	No	42.9% (21)		44.9% (22)	
**Location of practice**					
Europe	Yes	36.8% (21)	0.739	45.6% (26)	0.201
	No	33.9% (43)		56.7% (72)	
USA	Yes	25.6% (10)	0.191	59% (23)	0.472
	No	37.2% (54)		51.7% (75)	
**Type of Practice Setting**					
Inpatient/Hospital	Yes	35.9% (14)	0.852	53.8% (21)	1.000
	No	34.5% (50)		53.1% (77)	

Appendix I

MASCC Survey of the Utilization of Cryotherapy and Compression Therapy for the Prevention of Chemotherapy Induced Neuropathy

Dear MASCC Members, As a member of the Multinational Association of Supportive Care in Cancer (MASSC), you are invited to participate in the survey titled Cooling and Compression for Prevention of Chemotherapy Induced Neuropathy of the Neurological Complications Study Group of MASCC. Please complete this survey by 10/29/2021. The survey is about understanding practices around the globe on prevention of chemotherapy induced neuropathy using cryotherapy (cooling) and or compression of limbs. We are aiming to understand current patterns of use since there is a great deal of variability on use of these methodologies as we await more definitive data on their efficacy. The survey will take between 5-10 minutes to complete. We encourage you to answer all the questions as it will provide optimal data. However, your participation in this study is voluntary, and you may withdraw from the survey at any time. No identifiable information will be gathered, and the information you provide will remain completely anonymous. There is no reasonably foreseeable risk of discomfort or distress associated with your participation in this study. It is possible to respond using many different devices, e.g. computer, tablet or mobile phones. Please complete the questionnaire in one session (there is no way to continue at a later time). Please do not hesitate to contact Alexandre Chan, PharmD (a.chan@uci.edu) with any questions. Looking forward to receiving your responses.

What is your current role?

- RN
- Advanced Practice Provider (Nurse practitioner, Physician Assistant)
- Pharmacist
- Physician
- Other, please explain ______________________

How many years have you been practicing in your respective field?

- Less than a year
- 1-5 years
- 6-10
- More than 10 years

Where do you currently practice?

- Asia
- Australia
- Middle East
- Europe
- Canada
- USA
- Central America
- South America
- Africa
- Other

What is your predominant clinical practice setting?

- Outpatient oncology practice
- Inpatient/hospital
- University setting/research

- Other

Definitions

- Cryotherapy: Cooling the extremities by using ice packs, external devices or wearable garments to administer cold therapy while undergoing chemotherapy
- Compression therapy: Wearing stockings, sleeves or gloves to reduce the microvascular flow through compression while undergoing chemotherapy

1. In your clinical practice, do you currently see patients receiving taxanes and oxaliplatin?
    - Taxens Only
    - Oxaliplatin Only
    - Both
    - Neither
    - Unsure
2. Are you aware of data suggesting that distal extremity cryotherapy might decrease taxane CIPN troubles?
    - Yes
    - No
    - Unsure
3. Are you aware of data suggesting that distal extremity compression (such as tight surgical gloves) therapy might decrease taxane CIPN troubles?
    - Yes
    - No
    - Unsure
4. Are you aware of data suggesting that distal extremity cryo-compression (combining both of these items) therapy might decrease taxane CIPN troubles?
    - Yes
    - No
    - Unsure
5. Are you aware of data that distal extremity cryotherapy has been used with patients receiving oxaliplatin?
    - Yes
    - No
    - Unsure
6. Do **you** recommend distal extremity cryotherapy, compression therapy or both to your patients receiving **taxane** chemotherapy? If so, which one(s)?
    - Compression therapy only
    - Cryotherapy only
    - Both
    - Neither
    - Unsure

7. Do **others** in your practice use distal extremity cryotherapy, compression therapy or both in their patients receiving **taxane** chemotherapy? If so, which one(s)?
    - Compression therapy only
    - Cryotherapy only
    - Both
    - Neither
    - Unsure
8. Do **you** recommend distal extremity cryotherapy, compression therapy or both to your patients receiving oxaliplatin chemotherapy? If so, which one(s)?

- Compression therapy only
- Cryotherapy only
- Both
- Neither
- Unsure

9. Do **others** in your practice use distal extremity cryotherapy, compression therapy or both in their patients receiving **oxaliplatin** chemotherapy?
    - Compression therapy only
    - Cryotherapy only
    - Both
    - Neither
    - Unsure
10. If you, or others in your practice, are recommending cryotherapy, what technology is being used? (Please check all that apply)
    - Bag with ice
    - Commercial cooling gloves and socks
    - Other, please explain________________________________
    - N/A (Not recommending)
11. If you, or others in your practice, are recommending compression therapy, what technology is being used?
    - Compression socks
    - Compression bandaging
    - Other, please explain________________________________
    - Not recommending
12. If you, or others in your practice, are recommending compression therapy, how long before administration of taxanes/oxaliplatin do you initiate usage? (Please check all that apply)
    - One hour before
    - Half an hour before
    - Unsure
    - N/A
    - Not recommending
    - Other, please explain ________________________________
13. If you, or others in your practice, are recommending compression therapy, how long after administration of taxanes/oxaliplatin do you initiate usage? (Please check all that apply)
    - One hour before
    - Half an hour before
    - Unsure
    - N/A
    - Not recommending
    - Other, please explain ________________________________

14. If you are not recommending any of the above, please list ALL the reasons for not doing so (Please check all that apply)
    - Logistics (including manpower to change timely change cryotherapy devices)
    - Potential hazards (ice melting causes slips and falls)
    - Not enough evidence
    - Worried about complications
    - Patient discomfort with cooling
    - Worried about making treatment less effective
    - Other, please explain___________________________
    - N/A (recommending)

15. What adverse events have patients identified when receiving cryotherapy or compression therapy? (Please check all that apply)
    - Numbness/ tingling
    - Redness/ Irritation of skin
    - Blisters
    - Frostbites
    - Other, please explain___________________________
    - N/A (recommending)
